# *Toxoplasma* calcium-dependent protein kinases 3 mediates M1 macrophage polarization by targeting host Arginase-1

**DOI:** 10.1186/s13071-025-06799-8

**Published:** 2025-05-20

**Authors:** Ran An, Fang Liu, Niuniu Dai, Fangmin Li, Xingyun Liu, Haijian Cai, Lijian Chen, Jian Du

**Affiliations:** 1 Department of Biochemistry and Molecular Biology, School of Basic Medical Sciences, Anhui Medical University, Hefei, 230032 China; 2 The Research Center for Infectious Diseases, School of Basic Medical Sciences, Anhui Medical University, Hefei, 230032 China; 3 The Provincial Key Laboratory of Zoonoses of High Institutions of Anhui, Anhui Medical University, Hefei, 230032 China; 4 Department of Anesthesiology, The First Affiliated Hospital of Anhui Medical University, Hefei, 230032 China

**Keywords:** *Toxoplasma gondii*, CDPK3, Arginase-1, Macrophages polarization, Proliferation

## Abstract

**Background:**

*Toxoplasma gondii,* an obligate intracellular parasite, has developed sophisticated ways to manipulate host immunity, resulting in long-lasting infection and causing serious public health problems in humans and animals. *T. gondii* type II is the type most frequently associated with human diseases, but the mechanism remains unclear. *Toxoplasma* calcium-dependent protein kinase 3(CDPK3), a protein located on the *T. gondii* parasite periphery, is highly expressed in type II strains. Although *Tg*CDPK3 regulates parasite egress from host cells, calcium-based infiltration, and development of tissue cysts, the host target proteins that it modulates are still unclear.

**Methods:**

Firstly, mass spectrometry was used to analyze proteins that selectively bind to *Tg*CDPK3. Subsequently, GST (glutathione-s-transferase) pull-down, immunoprecipitation, and immunofluorescence assay were used to confirm the interaction and colocalization between *Tg*CDPK3 and Arginase-1. Western blotting and Argininaseactivity assays were performed to detect the relative levels of endogenous Arginase-1 and inducible nitric oxide synthase (iNOS) in a murine microglial cell line. Fluorescence activated cell sorting (FACS) assays and enzyme-linked immunosorbent assay (ELISA) analysis were performed to confirm the association of interaction between *Tg*CDPK3 and Arginase-1 within an M1/M2-polarized macrophage. Intracellular multiplication assays and plaque assays were performed to test whether the interaction between *Tg*CDPK3 and Arginase-1 affected intercellular parasite growth.

**Results:**

The interaction between *Tg*CDPK3 and Arginase-1 is functionally correlated and leads to a reduction in Arginase-1 activity, ultimately, contributing to the M1-biased phenotype of the host macrophages, which is related to restraining the proliferation of parasites.

**Conclusions:**

Our data showed that CDPK3 mediates M1 macrophage polarization by targeting host Arginase-1, which is beneficial to understanding the mechanism for long term latency establishment of less virulent strains of *Toxoplasma*.

**Graphical Abstract:**

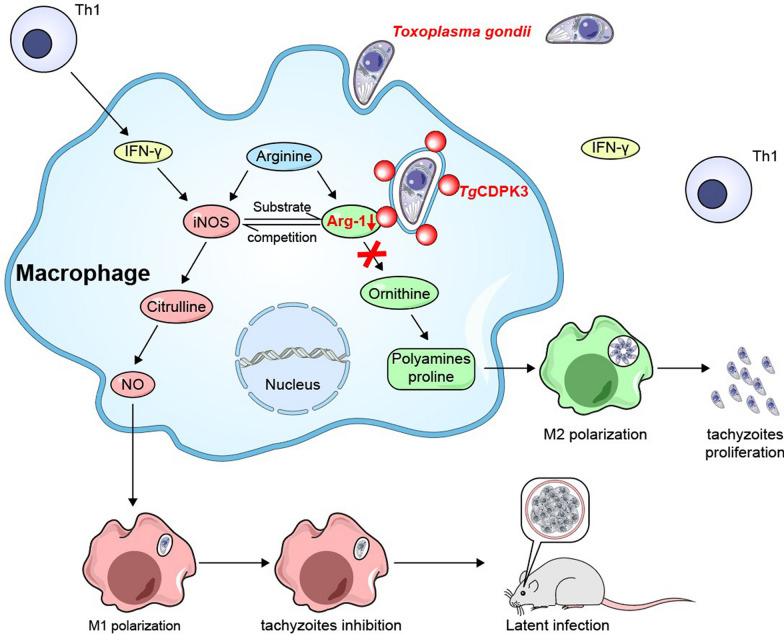

**Supplementary Information:**

The online version contains supplementary material available at 10.1186/s13071-025-06799-8.

## Background

As an apicomplexa protozoa, *T. gondii* can infect almost all warm-blooded animals, including humans, causing serious physical and mental illness. According to statistics, nearly 30% of the world’s population is chronically exposed and infected with *T. gondii* [1]. Early studies on the range of infection classified *T. gondii* into three genetic lineages (type I, type II, and type III), with the most commonly observed in North America and Europe [2]. Type II (mainly PRU and ME49) possesses moderate virulent in murine models, which are responsible for human toxoplasmosis and are associated with asymptomatic illness in immunocompetent individuals [3]. Generally, the invasion to host cells of *T. gondii* mainly depend on two mechanisms: the classic active penetration for virulent strain’s invasion of nonphagocytic cells, and a noncanonical invasion pathway when a virulent strain infected host macrophages [4–6]. Avirulent tachyzoites infect host macrophages via phagocytosis at the initial stage and form a parasitophorous vacuole (PV) subsequently through active penetration [6]. In this phase, the surface effectors of parasites may involve in the regulation of host intracellular signal transduction.

*Tg*CDPK3 is a characteristic member of calcium-dependent protein kinases, which is located in the periphery of the *T. gondii* and is considered to be an indispensable element for *T. gondii* parasites egress from host cells, calcium-based infiltration to control parasite vacuolar membranes, and tissue cyst development in the mouse brain [7, 8]. However, host proteins targeted by *Tg*CDPK3 and associated molecular mechanisms remain unclear. In our previous studies, we found *Tg*CDPK3 could activate the autophagy of host cells through the interaction with the Atg3 and Atg5, resulting in the recruitment of immune-related proteins to PV membrane subsequently, which was beneficial to establish the long-term latency in host macrophages [9]. Accordingly, considering that *Tg*CDPK3 plays an important role in the less virulent strain, proteomic analysis of proteins that selectively bind to *Tg*CDPK3 was performed by mass spectrometry. To our surprise, the host cytoplasmic Arginase-1, a urea recycling enzyme that can catalyze the hydrolysis of L-arginine to urea and L-ornithine, was found as a target protein of *Tg*CDPK3. The interaction between *Tg*CDPK3 and Arginase-1 led to the suppression of Arginase-1 activity. We used CDPK3-knockout type II ME49 (ME49_*Δcdpk3*_) and ME49 wild-type (ME49_*wt*_) to detect the enzyme activity of Arginase-1. We confirmed that *Tg*CDPK3 could suppress the activity of Arginase-1 and induced M1-biased activation, which is related to restraining the proliferation of parasites. Collectively, these results provide a new insight into the immune escape of type II strain and lay a foundation to elucidate the host’s immune response to *T. gondii* infection.

## Methods

### Cells and parasites

The HEK293T cell lines (CRL-3216), BV2 cell lines, Vero cell lines, and HFF cell lines (SCRC-1041) were stored in liquid nitrogen in our laboratory and cultured in DMEM (VivaCell, C3103-0500, China) supplemented with 10% FBS (VivaCell, C04001-500, China) and 1% penicillin/streptomycin/amphotericin (Procell, PB180121, China). The ME49_*Δcdpk3*_ strain was generated in our previous study and maintained by our laboratory [9]. The ME49_*wt*_ strain and ME49_*Δcdpk3*_ strain were maintained in HFF cells in the DMEM media. All cells and parasites were evaluated regularly for mycoplasma contamination. No mycoplasma contamination was observed.

### Reagents and plasmids

The primary antibodies Anti-iNOS (1:1000, cat no. 22226–1-AP), anti-Arginase-1(1:5000, cat no. 16001–1-AP), anti-β-actin (1:10,000, cat no. 66009–1-Ig), and anti-GAPDH (1:10,000, cat no. 60004–1-Ig) were obtained from Proteintech Group (China). Anti-SAG1 (1:1000, cat no. GTX36747) was obtained from GeneTex (USA). Anti-His (1:2000, cat no. CW0286M) and anti-GFP （green fluorescent protein） (1:1000, cat no. CW0086M) was obtained from Cowin Bio (China). The secondary HRP-conjugated antibodies were purchased from Proteintech Group (China). The full ORF encoding human Arginase-1 (GenBank no.: NM_000045.3) was achieved through quantitative reverse transcription polymerase chain reaction (RT-PCR) of the whole L02 cells RNA. The ORF of *Tg*CDPK3 (GenBank no.: XP002370358.1) was obtained through RT-PCR of the ME49 strain RNA. Full-length for human Arginase-1 was subcloned into the pET-28a (Novagen, 69,864, German) and pT7-FLAG-4 (Sigma-Aldrich, P9743, USA). Full-length for *Tg*CDPK3 was subcloned into a pEGFP-C2 vector (youbio, VT1108, China) and pGEX-6p-1 vector (youbio, VT1258, China). All constructs were confirmed through DNA sequencing.

### GST pull-down assay

*Tg*CDPK3-GST and GST proteins were conjugated to glutathione magnetic agarose beads (ThermoFisher, 78,601, USA). Purified Arginase-1-His was incubated with the glutathione magnetic agarose beads at 4 °C for 2 h. After being washed with PBS containing 1% triton X-100, the beads were subjected to 12% SDS-PAGE followed by immunoblot analysis.

### Immunoprecipitation assay and western blot analysis

The 293 T transient co-expressed Arginase-1 and *Tg*CDPK3 for 24 h were lysed in lysis buffer (50 mM HEPES, 2 mM EGTA, 150 mM NaCl, 1% Triton X-100, 1 mM phenylmethylsulfonyl fluoride) containing a protease inhibitor (Roche, 04693116001, USA). The cell lysates were incubated with Protein A + G affinity gel (Beyotime, P2055, China) coupled with anti-GFP antibody for 4 h. The immunoprecipitants were eluted and separated using 10% SDS-PAGE followed by immunoblot analysis.

### Immunofluorescence assay

The cells plated onto glass coverslips were fixed with 4% paraformaldehyde. After being permeabilized with 0.1% triton X-100 and blocked with 5% BSA, the cells were incubated with anti-Arginase-1. Rhodamine-conjugated goat anti-mouse immunoglobulin (Ig)G and DAPI dye (Beyotime, C1002, China) were used for antigen and DNA visualization. The images were captured by a laser scanning confocal microscope (ZEISS, LSM880 + airyscan, German) with identical acquisition parameters for each experiment. The following antibodies were used at the specific dilutions: anti-Arginase-1 mAb (1:100; Proteintech, 16,001–1-AP, China), anti-CDPK3 (1:100, taopu, China), Rhodamine-conjugated goat anti-mouse IgG (1:400, ThermoFisher, 31,660, USA), and FITC-conjugated goat anti-rabbit IgG (1:400; ThermoFisher, 65–6111, USA).

### Argininase activity assays

After being transfected with *Tg*CDPK3-GFP and GFP or infected with ME49_*Δcdpk3*_ and ME49_*wt*_ tachyzoites, the cells were lysed in lysis buffer (10 mM Tris–HCl pH 7.4, 1 μM leupeptin, 1 μM pepstatin, and 0.4% triton X-100). The Arginase-1 activity was determined using the kit components according to the manufacturer’s instructions (boxbio, AKAM022M, China).

### Nitrite oxide production

Nitrite was measured by using Griess reagent (Beyotime, S0024, China). Briefly, 50μL equal volumes of cells culture supernatants and Griess reagent were mixed and incubated. Absorbance was measured at 540 nm in a microscope plate reader (ThermoFisher, Multiskan^™^ SkyHigh, USA). The nitrite value was obtained from a calibrated sodium nitrite standard curve ranging from 0 to 60 μM.

### ELISA analysis

The cell culture supernatants of BV2 cells infected with ME49_*Δcdpk3*_ and ME49_*wt*_ tachyzoites at a MOI (multiplicity of infection) of 1 were analyzed using an ELISA for mouse tumor necrosis factor-alpha (TNF)-α (Proteintech, KE10002, China), interleukin (IL)-6 (Proteintech, KE10007, China), transforming growth factor beta (TGF-β) (Proteintech, KE10005, China), and IL-10 (Proteintech, KE10103, China). The absorbance was detected at OD450 nm in a microscope plate reader (ThermoFisher, Multiskan™ SkyHigh, USA). The experiments were repeated three times, and the data represent the mean of five wells ± SEM.

### Flow cytometry analysis

BV2 cells challenged with ME49_*Δcdpk3*_ and ME49_*wt*_ tachyzoites were stained with anti-F4/80(1:100; Biolegend, 123,137, USA), anti-CD86(1:200; Biolegend, 159,216, USA), and anti-CD206(1:100; Biolegend, 141,706, USA) antibodies. Then, the cells were washed and resuspended for flow cytometry (Beckman Coulter, CytoFLEX S, USA).

### *T. gondii* intracellular multiplication assays

After being challenged with ME49_*Δcdpk3*_ and ME49_*wt*_ tachyzoites at a MOI of 3, Vero cells were stained with Giemsa reagent (Beyotime, C0131, China). The numbers of tachyzoites were counted in 200 host cells, and an average was determined.

### Quantitative Real-Time PCR

Total RNA was extracted using RNA simple Total RNA Kit (TIANGEN, China). Then the RNA was reverse transcribed to cDNA using the Advantage RT-for-PCR Kit (TaKaRa, 639,505, Japan). With the SYBR Green Pro Taq HS (Accurate Biology, AG11759, China), RT-PCR was performed to examine the expression level of *Tg*SAG1, using β-actin as a relative quantification to evaluate transcript abundance. Reactions were run on the Roche LC480II system (Roche, LightCycler480II, USA). The primer sequences of *Tg*SAG1 and β-actin are listed in Supplementary Table 1. Each sample was measured three times. Data represent three separate experiments.

### Plaque assays

Cells were plated in six-well plates. Freshly harvested ME49_*Δcdpk3*_ and ME49_*wt*_ tachyzoites (1000 per strain) were added to plates. After growth for 10 days, the cells were fixed with 4% paraformaldehyde and stained with 0.1% crystal violet. The plates were subsequently scanned with scanner (MICROTEK, FileScan 1710XLplus, China). All tachyzoites were tested three times and each with three internal replicates.

### Statistical analysis

Statistical analysis and graphics were conducted by GraphPad Prism. Data are presented as the mean ± SEM. Differences between groups were identified using one-way analysis of variance (ANOVA), including antibody levels, cytokine levels, arginase activity levels, NO (nitric oxide) levels, and cell percentages. Two-way ANOVA is applied to assess differences in number of vacuoles containing 2, 4, 8, or 16 parasites. Here, **P < 0.01 and ***P < 0.001 represented varying degrees of significance.

## Results

### *Tg*CDPK3 directly interacts with Arginase-1

**Fig. 1:**
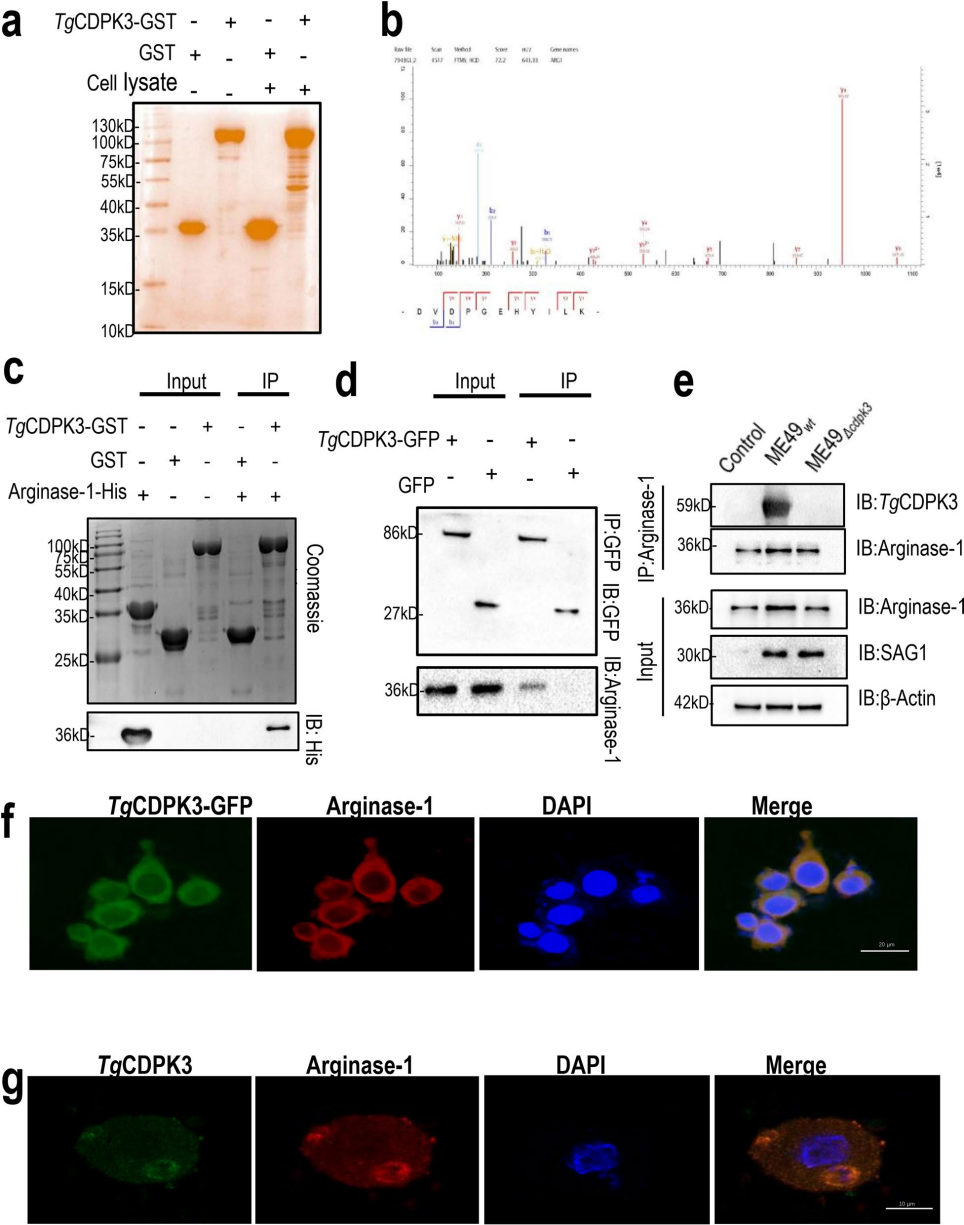
Identification of Arginase-1 as a *Tg*CDPK3-interacting host protein. **a** Purified GST-*Tg*CDPK3 was incubated with macrophage lysates, and the sodium dodecyl sulfate–polyacrylamide gel electrophoresis (SDS–PAGE) gel was silver-stained to determine proteins interacting with *Tg*CDPK3. **b** Protein spectrum analysis Arginase-1 is the interacting protein of *Tg*CDPK3. **c** Confirmation of arginase-1 binding to *Tg*CDPK3 using in vitro pull-down assays. *Tg*CDPK3-GST purified on glutathione beads was used as an affinity matrix for absorbing Arginase-1-His fragment for SDS–PAGE. After stained with Coomassie Brilliant Blue (upper panel), the gel was blotted with anti-His antibody (lower panel). **d** Verification of the interaction of *Tg*CDPK3 with Arginase-1 through immunoprecipitation. HEK 293 T cells transfected with *Tg*CDPK3-GFP or control GFP vector were immunoprecipitated with anti-GFP. Starting fractions (input) and immunoprecipitates (IP) were analyzed and blotted with anti-GFP and anti-Arginase-1 antibodies. **e** Using ME49_*wt*_ or ME49_*Δcdpk3*_ strains to determine the interaction of *Tg*CDPK3 with Arginase-1. BV2 cells were infected with the indicated parasites at MOI of 3. Immunoprecipitation (IP) of Arginase-1 from infected cell lysates was detected with rabbit polyclonal *Tg*CDPK3 antibody. **f** BV2 cells grown on glass coverslips were transfected with *Tg*CDPK3-GFP (green) for 24 h. Double immunofluorescence was performed using anti-Arginase-1 (red) and DAPI (DNA, blue). A distinctive pattern of *Tg*CDPK3 and Arginase-1 colocalization was observed in the cytoplasm. **g** BV2 cells grown on glass coverslips were infected with the ME49_*wt*_ strain at MOI of 1 for 12 h. Double immunofluorescence was performed using rabbit polyclonal anti-*Tg*CDPK3 (green) and anti-Arginase-1 (red) antibodies. Distinctive Arginase-1 and *Tg*CDPK3 localization was observed around the parasite

To identify host proteins targeted by *Tg*CDPK3, we performed proteome analysis. Specifically, *Tg*CDPK3-GST was used to isolate proteins interacting with active *Tg*CDPK3 from mouse leukemia cells of monocyte macrophage cell line extracts (Fig. 1A). To distinguish putative *Tg*CDPK3-binding proteins from nonspecific binding, we performed proteomic analysis of proteins that selectively bind to TgCDPK3 using mass spectrometry. Interestingly, Arginase-1, a cytosolic enzyme, was identified (Fig. 1B). To demonstrate a direct interaction between *Tg*CDPK3 and Arginase-1, we performed GST pull-down assays in vitro using recombinant *Tg*CDPK3-GST and Arginase-1-His expressed in *E. coli*. The results showed that *Tg*CDPK3, but not GST, was able to pull down Arginase-1-His, demonstrating that *Tg*CDPK3 physically bound to Arginase-1(Fig. 1C). To further confirm this result, *Tg*CDPK3-GFP or GFP vector was transfected into 293 T cells and performed immunoprecipitation assay using an anti-GFP antibody. The result suggested that *Tg*CDPK3 indeed interacted with Arginase-1 in vivo (Fig. 1D). Consistent results were found in murine microglial cells infected with the ME49_*wt*_ or ME49_*Δcdpk3*_ strains, confirming the interaction between endogenous *Tg*CDPK3 and Arginase-1 (Fig. 1E). In addition, we observed that Arginase-1 colocalized with *Tg*CDPK3 in the cytoplasm, which was addressed the physiological relevance of interaction between *Tg*CDPK3 and Arginase-1 in host cells (Fig. 1F). It has been reported that *Tg*CDPK3 controls calcium-dependent permeabilization in the PV membrane during parasite egress and tissue cysts formation, mainly located at the periphery of the parasite [8]. Here, we observed the colocalization of *Tg*CDPK3 and Arginase-1 in the periphery of ME49_*wt*_ strain in infected murine microglial cells (Fig. 1G). Therefore, these results indicate that *Tg*CDPK3 interacts with Arginase-1 both in vitro and in vivo directly.

### *Tg*CDPK3 influences the activity and expression of Arginase-1

**Fig. 2:**
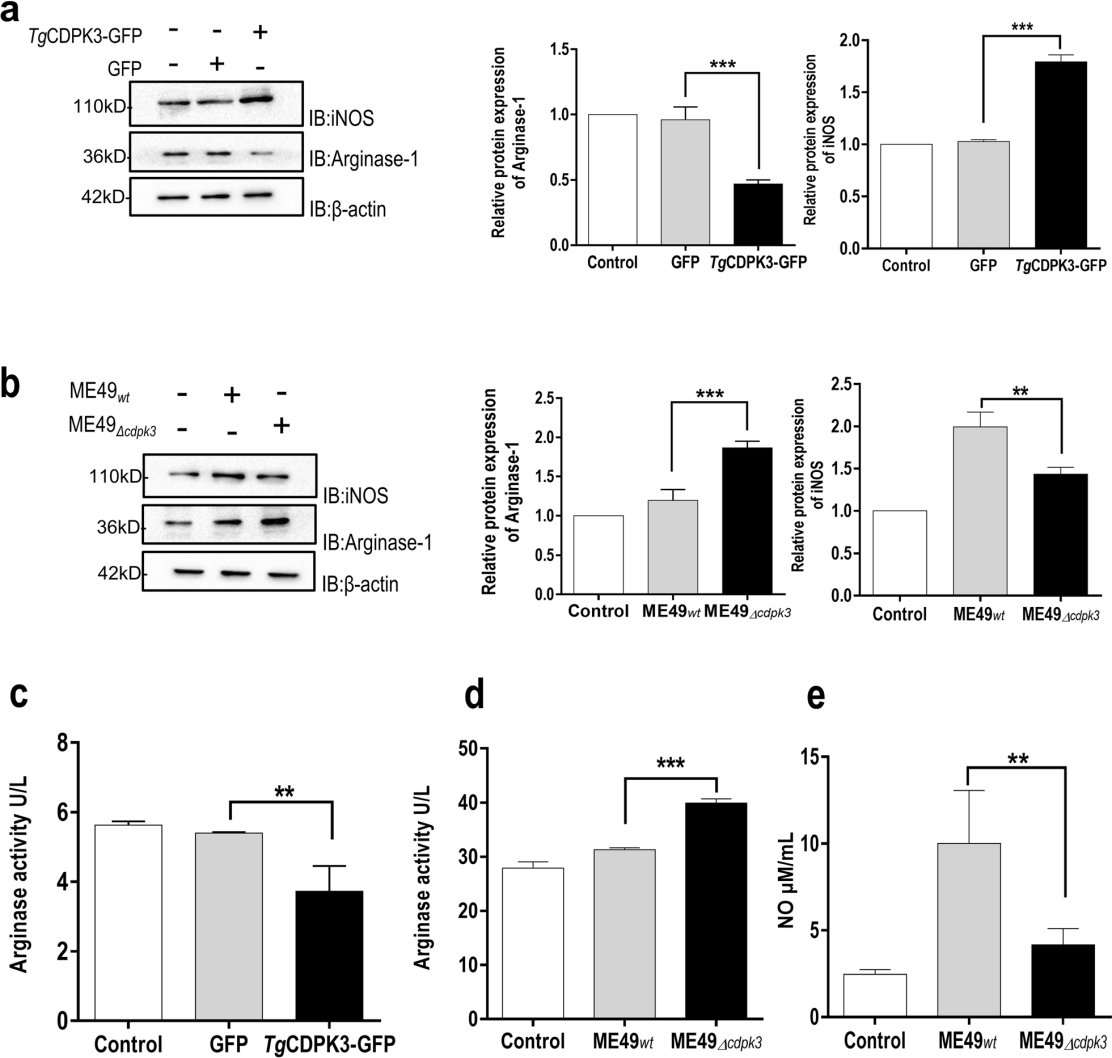
*Tg*CDPK3 interacts with Arginase-1 and influences the activity and expression of Arginase-1 and iNOS. **a** BV2 cells were transfected with *Tg*CDPK3-GFP or control GFP vector. At 24 h after transfection, the cells were lysed and subjected to western blotting (IB) with anti-Arginase-1 and anti-iNOS. The Arginase-1 and iNOS expression levels were normalized against β-actin. (***P < 0.001 via one-way ANOVA). Data are presented as mean ± SEM. **b** BV2 cells infected with ME49_*wt*_ or ME49_*Δcdpk3*_ strains at MOI of 3 for 12 h were analyzed and blotted with anti-Arginase-1 and anti-iNOS antibodies. The Arginase-1 and iNOS expression levels were normalized against β-actin. (**P < 0.01, ***P < 0.001 via one-way ANOVA). Data are presented as mean ± SEM. **c** After being transfected with *Tg*CDPK3-GFP and GFP, BV2 cells were lysed to determine the Arginase-1 activity. (**P < 0.01 via one-way ANOVA). Data are presented as mean ± SEM. **d** After being infected with ME49_*wt*_ or ME49_*Δcdpk3*_ strains at MOI of 3, the BV2 cells were lysed to determine the Arginase-1 activity. (***P < 0.001 via one-way ANOVA). Data are presented as mean ± SEM. **e** BV2 cells infected with ME49_*wt*_ or ME49_*Δcdpk3*_ strains at MOI of 3. The cells culture supernatants were used to detect the production of NO. (**P < 0.01 via one-way ANOVA). Data are presented as mean ± SEM

Generally, Arginase-1 competes with iNOS for the common substrate L-arginine, hydrolyzing L-arginine to urea and L-ornithine. L-ornithine is a precursor for the synthesis of L-glutamine, L-proline, and polyamines via the ornithine decarboxylase (ODC) pathway, promoting parasite replication by providing the polyamines needed for cell division [10]. As we mentioned above, Arginase-1 is a *Tg*CDPK3-interacting protein, thus we focus on the functional relevance of this interaction. *Tg*CDPK3-GFP was transfected and overexpressed in murine microglial cell line BV-2 and the relative levels of endogenous Arginase-1 and iNOS were detected via western blotting. The results revealed that overexpression of *Tg*CDPK3 reduced the level of Arginase-1 and enhanced the level of iNOS significantly (Fig. 2A). Next, we infected murine microglial cells with ME49_*wt*_ or ME49_*Δcdpk3*_ parasites and found that ME49_*Δcdpk3*_ significantly induced a high level of Arginase-1 and decreased the level of iNOS compared with ME49_*wt*_ strain (Fig. 2B). To determine whether the interaction between *Tg*CDPK3 and Arginase-1 affected the activity of Arginase-1 and iNOS, we overexpressed *Tg*CDPK3 and found that *Tg*CDPK3 reduced the activity of Arginase-1 (Fig. 2C). Meanwhile, microglial cells infected with ME49_*wt*_ or ME49_*Δcdpk3*_ strains were lysed to perform Arginase-1 activity assays. The result showed that *Tg*CDPK3 knockout parasites showed a higher level of Arginase-1 activity (Fig. 2D). Correspondingly, the level of NO in cells infected with ME49_*Δcdpk3*_ strain was significantly lower than that in ME49 _*wt*_ strain (Fig. 2E). Together, these data showed that *Tg*CDPK3 could inhibit the expression and activity of Arginase-1, which led to a shift in L-arginine metabolism, a decrease in polyamine production, and an increase in NO production.

### The interaction of* Tg*CDPK3 with Arginase-1 contributes to the M1-biased phenotype of the host macrophages

**Fig. 3:**
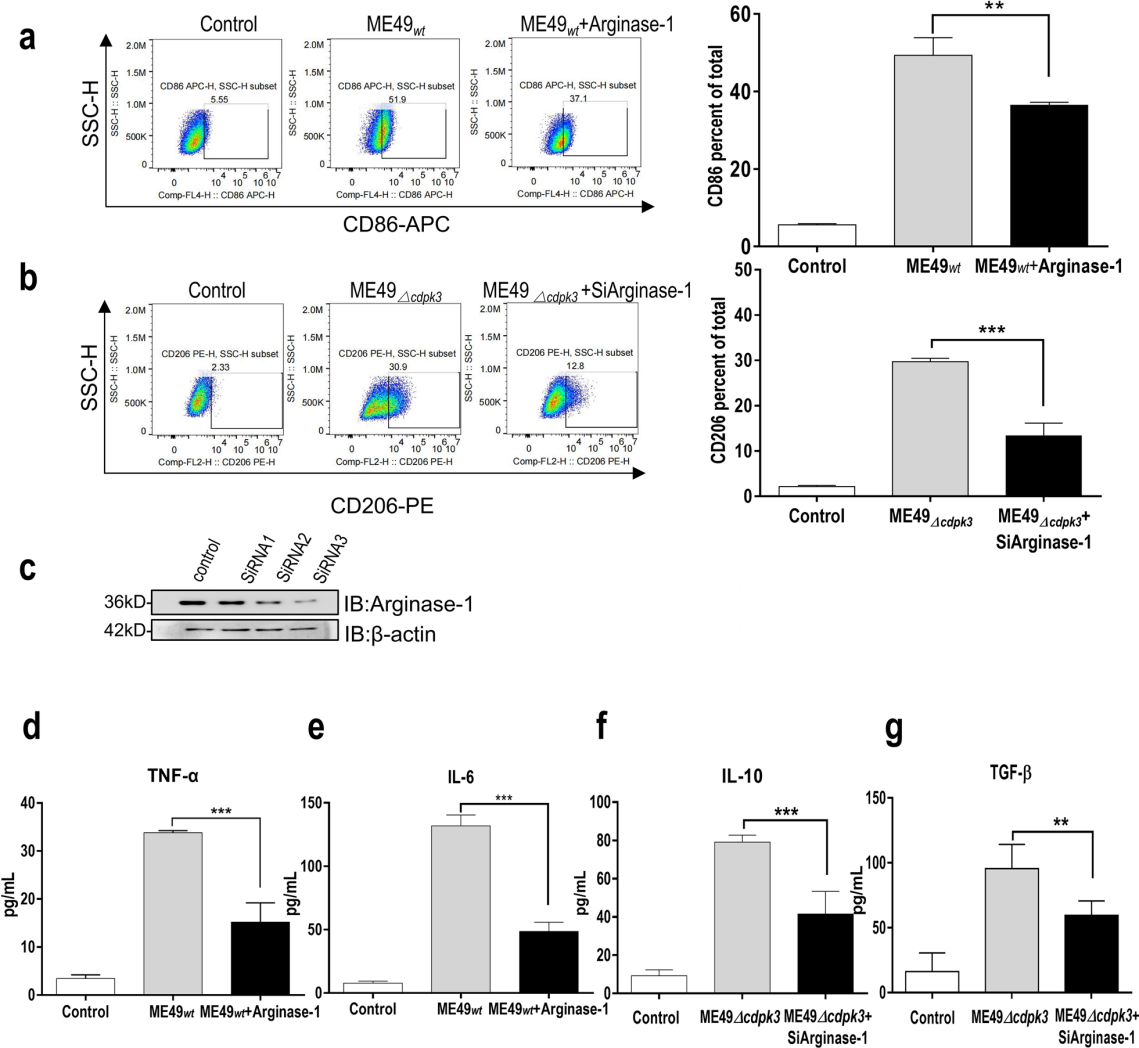
The interaction between *Tg*CDPK3 and Arginase-1 contributes to the M1-biased phenotype of the host macrophages. **a**–**b** BV2 cells infected with ME49_*wt*_ or ME49_*Δcdpk3*_ strains for 24 h were stained for CD86 and CD206. Histograms depict the percentage of positively stained CD86 and CD206 cells analyzed. Indicated values are the means ± SD of triplicates compared with the ME49_*wt*_ control. (**P < 0.01, ***P < 0.001 via one-way ANOVA). Data are presented as mean ± SEM. **c** BV2 cells were transfected with either control siRNA ( small interfering RNA) or siRNA targeting Arginase-1 for 24 h. The cell lysates were analyzed and blotted with anti-Arginase-1 antibodies. Arginase-1 expression levels were normalized against β-actin. **d–g** BV2 cells were transfected with Arginase-1 or Arginase-1 siRNA, then infected with the indicated ME49_*wt*_ or ME49_*Δcdpk3*_ strain at MOI of 3 for more than 12 h. Concentrations of TNF-α **(d)**, IL-6**(e)**, IL-10 **(f)**, and TGF-β **(g)** in the culture supernatants were measured through ELISA. (**P < 0.01, ***P < 0.001 via one-way ANOVA). Data are presented as mean ± SEM

**Fig. 4:**
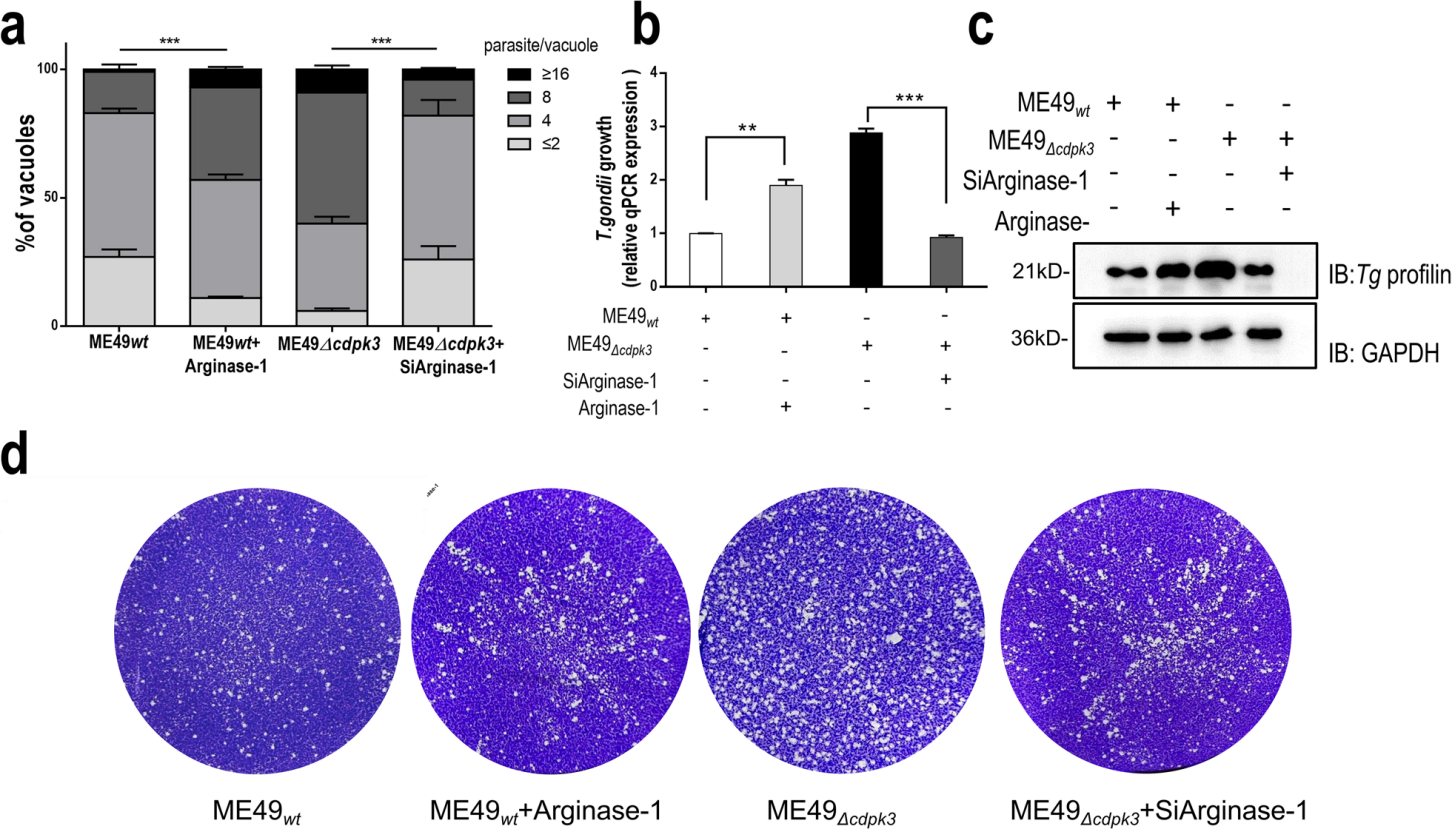
The interaction of *Tg*CDPK3 with Arginase-1 influences the intracellular proliferation of *T. gondii.* BV2 cells were pre-transfected with control or Arginase-1 siRNA before being infected with ME49_*wt*_ or ME49_*Δcdpk3*_ strains. **a** At 36 h post-infection, 200 vacuoles were inspected to determine the number of vacuoles containing 2, 4, 8, or 16 parasites. Error bars indicate standard deviations of the measured values. (***P < 0.001 via two-way ANOVA). Data are presented as mean ± SEM. **b** Parasite growth was also quantified by qPCR of genomic DNA using the surface antigen (SAG)1 primers and normalized against β-actin. (**P < 0.01, ***P < 0.001 via one-way ANOVA). Data are presented as mean ± SEM. **c** Total infected cell proteins were analyzed and blotted with anti- *T. gondii* profilin. The profilin expression levels were normalized against GAPDH. **d** Plaque formation of ME49_*wt*_ or ME49_*Δcdpk3*_ parasites, in the presence or absence of Arginase-1

Pathogen-associated molecular patterns (PAMPs), in combination with interferon-gamma (IFN-γ), polarize macrophages to an M1 phenotype featuring NO and ROS production, which help to kill microbial pathogens. Previous research stated that *T. gondii* type I and type III strains induce macrophages displayed as an alternatively activated phenotype (termed M2), whereas type II strains induce a classically activated phenotype (termed M1) [11]. Unlike M1 macrophages express cytokines and chemokines that activate antimicrobial activity in host cells, M2 macrophages can down-regulate Th1 type responses and promote wound repair through secreting antiinflammatory molecules [12]. To determine the association of interaction between *Tg*CDPK3 and Arginase-1 with a M1/M2 macrophages polarized phenotype, we overexpressed Arginase-1 in macrophages infected with ME49_*wt*_ parasites. FACS assay results showed that macrophages infected ME49_*wt*_ elicited a higher level of the M1-associated markers (CD86), whereas Arginase-1 overexpression reduced the increase of CD86 (Fig. 4A). In addition, the M2 signature (CD206) was clearly observed in mouse macrophages infected with ME49_*Δcdpk3*_ strain, which is probably because this strain cannot inhibit the Arginase-1. Meanwhile, deficiency of Arginase-1 reduced the expression of CD206 when infected with ME49_*Δcdpk3*_ strain (Fig. 3B and C). Next, we detected M1-associated signature cytokines (TNF-α and IL-6) and M2-associated signature cytokines (TGF-β and IL-10). Consistent with the above findings, macrophages infected ME49_*wt*_ induced high secretions of TNF-α and IL-6 compared with the uninfected group, whereas cells overexpressing Arginase-1 greatly inhibited the TNF-α and IL-6 levels (Fig. 3D and E). In addition, macrophages infected ME49_*Δcdpk3*_ induced higher secretions of TGF-β and IL-10, whereas deficiency of Arginase-1 reduced the production of TGF-β and IL-10 (Fig. 3F and G). Taken together, *Toxoplasma* CDPK3 contributed to the host macrophage's M1-biased phenotype through inhibiting the host Arginase-1.

### The interaction of *Tg*CDPK3 with Arginase-1 influences the intracellular proliferation of *T. gondii*

Considering that *Tg*CDPK3 reduced Arginase-1 activity and expression to promote NO production, and the existence of NO could help to kill microbial pathogens, we hypothesized that *Tg*CDPK3 may limit the proliferation of parasites through Arginase-1. First, we assessed whether *Tg*CDPK3 and Arginase-1 interaction affects the growth of intercellular parasites. Results showed that eight or more parasites accounted for a higher proportion in the vacuoles of ME49_*Δcdpk3*_ strain compared with ME49_*wt*_ strain, and Arginase-1 knockdown greatly reduced the number of parasites per vacuole. Consistently, Arginase-1 overexpression led to an increase in the numbers of parasites per vacuole in ME49_*wt*_ infection group. These results suggested that *Tg*CDPK3-dependent proliferation limitation was partly dependent on Arginase-1 (Fig. 4A). Next, the parasite growth results were also confirmed by qPCR of the genomic DNA and western-blotting analysis (Fig. 4B and C). Moreover, whether the interaction between *Tg*CDPK3 and Arginase-1 affect growth of parasites was verified by plaque assays. After infection for 10 days, the size of plaques and the number of plaques per well were calculated. Consistent with our previous results, the number and size of ME49_*Δcdpk3*_ strain plaques were larger than that of the ME49_*wt*_ strain, and deficiency of Arginase-1 significantly decreased in the number and size of plaques formed caused by ME49_*Δcdpk3*_ strain infection, indicating that the deficiency of Arginase-1 results in resistance to the increase of plaques formation by ME49_*Δcdpk3*_ (Fig. 4D). Taken together, all these results confirm that *Tg*CDPK3 can inhibit the growth of the parasites by inhibiting the host Arginase-1.

## Discussion

*T. gondii* could invade all nucleated cells, both nonphagocytic and phagocytic cells, with the actively invade mechanism carried out by the parasite to host cells called active invasion. *T. gondii* has established a strategy for acquisition and entry into host cells to ensure its transmission in host. This mechanism involves the assembly of temporary structures called mobile junctions, which consist of parasite and host cell proteins that form a complex underneath the host cell plasma membrane [13]. To combat this invasion of *T. gondii*, macrophage, which is the cell type preferentially infected by the parasite in vivo, plays an essential role in the early immune response [14, 15]. To combat macrophages, *T. gondii* evolved various methods and mechanisms to resist macrophage clearance and utilize their migratory activities to promote parasites dissemination [15]. The mechanism of *T. gondii* ingress into macrophage has been characterized as phagocytosis, particularly in macrophages of less virulent tachyzoites infections, where the cells surrounding the parasite extend pseudopods and subsequently form typical phagocytic vesicles and form PV through active penetration from within the phagosome. In this phase, the surface effectors of parasites may interact with the host cytoplasmic proteins to regulate cellular immunity [4, 6]. CDPKs have been identified in protozoans and plants, and may control microneme secretion, cytoskeletal dynamics, and regulation of motility complexes, related to calcium-dependent processes, thereby influencing gliding motility, cell invasion, and expulsion [16]. Thus, this study utilized mouse macrophages infected with *T. gondii* to investigate the targets regulated by *Tg*CDPK3 and the underlying mechanisms.

Our previous investigations demonstrated that *Tg*CDPK3 was highly expressed in ME49 and *Tg*Ctwh6 strains, which belong to less virulence strains [9]. Considering this characteristic, it is worth looking into the *Tg*CDPK3 interacting proteins of host cytoplasm and its immunomodulatory effect. Here, we demonstrate that Arginase-1, an integral and vital part of the hepatic urea cycle, is a specific substrate of the *Tg*CDPK3. The interaction between *Tg*CDPK3 and Arginase-1 was further verified through co-immunoprecipitation, GST pull-down, and immunofluorescence assays.

Arginase-1 can be induced in many cell types, such as macrophages and endothelial and epithelial cells, which is essential for L-arginine metabolism and macrophage polarization [17]. As an essential amino acid, L-arginine can not only modulate the host cellular immune response during pathogen infection, but also as a shared substrate for iNOS and Arginase-1 [18]. Arginine has two main metabolic pathways: (1) Arginase hydrolyzes arginine to urea and ornithine, and ornithine decarboxylase (ODC) catalyzes the enzymatic decarboxylation of L-ornithine in putrescine, which is a metabolic process to synthesize polyamines. Polyamines are essential metabolites in eukaryotes that participate in a variety of proliferative processes [19]. A mechanism that impairs antimicrobial defense in vivo by Arginase-1 may lead to the generation of ornithine and via the ODC pathway to the synthesis of polyamines [19]; and (2) Arginine produces NO in macrophages by NO synthase (NOS II). NO is a core constituent of mouse macrophage innate immunity and is an efficacious antimicrobial agent, notably against intracellular pathogens [20]. Considering the effect of Arginase-1 on the control of parasite growth, we explored the molecular mechanisms between *Tg*CDPK3 and Arginase-1. Our study revealed that *Tg*CDPK3 could inhibit the expression and activity of Arginase-1 and increase the production of NO (Fig. 2). This suggested that *Tg*CDPK3 led to a shift in L-arginine metabolism, resulting in decreased polyamine production and increased nitric oxide (NO) production through the inhibition of Arginase-1.

*T. gondii* induces distinct polarization of host macrophages in a genotype-dependent manner, including both classically activated macrophages (M1) and alternatively activated macrophages (M2). It has been reported that, during the early stage of *T. gondii* type I and III infection, mouse macrophages tend to M2 phenotype through the STAT3/STAT6 pathways by rhoptry protein 16, while type II infected macrophages tend to M1 phenotype via dense granule protein 15 phosphorylated NF-κB [21]. *T. gondii* employs strategies for intracellular parasitism that include manipulating immune cell responses to prevent overreaction and modifying macrophage phenotypes to dampen the inflammatory response, thereby reducing harm to the host [22]. Arginase-1, a marker of alternative activation in murine macrophages, exacerbates disease by inhibiting the elimination of parasites by M1 macrophages and by promoting parasite multiplication [23]. Therefore, we explored whether changes in macrophage phenotype, induced by the inhibition of Arginase-1 due to *Tg*CDPK3, occur. As expected, we found that ME49_*wt*_ strain-infected macrophages developed obvious M1 polarization accompanied by increased secretion of TNF-α and IL-6, which would inhibit the growth of parasites. The M2 signature (CD206) was clearly noted in macrophages infected with ME49_*Δcdpk3*_ strain with increased secretion of TGF-β and IL-10 cytokines. TGF-β and IL-10 are potent suppressors of macrophage and dendritic cell activities. When mice deficient in these cytokines are infected with *T. gondii*, they exhibit exacerbated inflammation characterized by increased production of IL-12 and TNF-α, as well as a lethal CD4 + T cell response [24]. Furthermore, the deficiency of Arginase-1 partially reduced the expression of CD206 in mice infected with the ME49_*Δcdpk3*_ strain (Fig. 3). All of these findings indicate that the interaction between TgCDPK3 and Arginase-1 contributes to the M1-biased phenotype of host macrophages.

It is well known that M1 macrophages have an improved presentation of antigens and the ability to eliminate intracellular pathogens, which is attributed to the release of NO and the synthesis of peroxynitrite, a derivative of the NO reaction [25]. Since the activity and expression of Arginase-1 are correlated with increased pathogen loads in infectious diseases [26–30], we found that ME49_*Δcdpk3*_ strain promotes the proliferation of *T. gondii* more than the ME49 wild-type strain in macrophages. Moreover, the deficiency of Arginase-1 resulted in resistance to the increase of plaque formation caused by ME49_*Δcdpk3*_, suggesting that the increased expression of CDPK3 in less virulent inhibited Arginase-1 reduces parasite burdens in the host cell to prevent killing its host, thus enabling replication and dissemination before tissue cysts are formed.

## Conclusions

According to our results, *Tg*CDPK3 could interact with Arginase-1, which could affect arginine metabolism by decreasing the activity and expression of Arginase-1, producing more NO and contributing to the M1-biased phenotype of the host macrophages, ultimately resulting in inhibition of parasites proliferation. This may provide new insight into the function of the *Tg*CDPK3 protein in immune escape of the parasites in the host.

## Supplementary Information

Supplementary material 1.

## Data Availability

No datasets were generated or analyzed during the current study.

## Abbreviations

*Tg*CDPK3: *Toxoplasma* calcium-dependent protein kinases 3
iNOS: Inducible nitric oxide synthase
BV2: Murine microglial cell line
HFF: Human foreskin fibroblast
DMEM: Dulbecco’s modified eagle medium
FBS: Fetal bovine serum
DNA: Deoxyribonucleic acid
RNA: Ribonucleic acid
RT-PCR: Quantitative reverse transcription polymerase chain reaction
FACS: Fluorescence activated cell sorting
ELISA: Enzyme linked immunosorbent assay
SDS–PAGE: Sodium dodecyl sulfate–polyacrylamide gel electrophoresis
PBS: Phosphate buffered saline
